# Photon-number-resolving detection enables single-photon LiDAR approaching the standard quantum limit

**DOI:** 10.1038/s41377-025-01880-4

**Published:** 2025-05-22

**Authors:** Feihu Xu

**Affiliations:** Hefei National Research Center for Physical Sciences at the Microscale and School of Physical Sciences, University of Science and Technology of China, Hefei, 230026 China

**Keywords:** Imaging and sensing, Single photons and quantum effects

## Abstract

A photon-number-resolving LiDAR approach and an active photon-number-filtering algorithm are proposed and demonstrated. This opens a new avenue for the development of single-photon LiDAR and relevant techniques to scientific study and real-world applications.

Single-photon light detection and ranging (LiDAR) is an optical imaging and sensing technique that combines single-photon detection, computational imaging algorithms, and related techniques^1^. It actively emits laser pulses from the system to the object and exploits the single-photon detectors to record the time-of-flight of echo photons, where the time-of-flight measurements are processed to obtain 3D information. Single-photon LiDAR can achieve exceptional potential for precise imaging and remote sensing through its picosecond-level temporal resolution and single-photon sensitivity, attracting widespread attention in research and applications^2^. Recent developments have witnessed remarkable progress in LiDAR techniques and imaging algorithms. The photon-efficient imaging algorithms enable the processing of LiDAR data to precisely reconstruct the 3D image using as low as one photon per pixel^3,4^. The advanced single-photon LiDAR systems can deliver detailed 3D images at kilometer range^5^, obtain the image of targets tens or hundreds of kilometers away^6,7^, or reconstruct the targets that are not in the line-of-sight^8,9^. These progresses provide exciting new prospects for the widespread LiDAR applications.

The detectors in the previous single-photon LiDAR systems can only distinguish whether there are photons or not (i.e., threshold detection^2^), but cannot resolve the exact number of photons. This makes it difficult to accurately extract signal photons flooded by the strong noise photons. In addition, the threshold detection will lose the statistical information of photon numbers, which prevents the LiDAR from reaching the standard quantum limit^10^ (SQL) set by the photon-number statistics.

In a paper in *Light: Science & Applications*^11^, Haochen Li et al., propose a photon-number-resolving (PNR) single-photon LiDAR and show the capability to approach the SQL of the amplitude measurement of the light field. To do so, they develop a superconducting nanowire single-photon detector (SNSPD) array to realize PNR detection for up to 16 photons through spatial multiplexing, and design an active photon number filter (APNF) to adaptive select the effective photon number content and construct temporal gate (see Fig. 1). The APNF is design to adaptive select the effective photon number content and construct temporal gate to filter out noise events according to the statistic distribution of photon numbers.

**Fig. 1:**
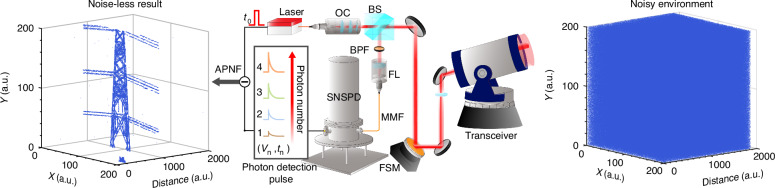
Schematic diagram of the proposed method. Schematic diagram of the LiDAR system with photon-number-resolving (PNR) detection. The data is obtained by the transceiver, detected by the superconducting nanowire single-photon detector (SNSPD) array, and processed by the active photon number filter (APNF) algorithm

Tested in the outdoor experiments, the proposed LiDAR system is capable to reconstruct the complex structure of a distributed pylon nearly noise-free over a standoff distance of 900 meters. The photon statistic measuring capability of the LiDAR is quantified by comparing the Fisher information of detection with the quantum Fisher information determined by the quantum fluctuation of coherent light. The results indicate that the proposed LiDAR can approach the SQL of the amplitude measurement of the light field within a large dynamic range, enabling faster and more accurate target measurement and materials identification in various scenarios.

Note that the single-photon LiDAR is believe to be particularly useful in the photon-starved situation with much less than one photon detection (per pulse period). This is indeed the scenario in the long-range target detection^3–7^, biology imaging^12^, non-line-of-sight imaging^8,9^ and so forth^1,2^. The proposed PNR approach will lose its advantage, but it may be valuable in the high-flux case^13^.

Recently, the emerging technologies such as single-photon avalanche diode, quantum light source, integrated photonics, and machine learning have been accelerating the advancement of single-photon LiDAR^12–16^. Overall, the successful integration of two advanced technologies, SNSPD array and the tailored computational imaging algorithm, has made it possible to realize target reconstruction approaching the SQL in the daytime. In the future, the PNR detection is expected to provide a new perspective for optical imaging and sensing.

## References

[1] Altmann, Y. et al. Quantum-inspired computational imaging. *Science* **361**, eaat2298 (2018). 30115781 10.1126/science.aat2298

[2] Hadfield, R. H. et al. Single-photon detection for long-range imaging and sensing. *Optica* **10**, 1124–1141 (2023).

[3] Kirmani, A. et al. First-photon imaging. *Science* **343**, 58–61 (2014). 24292628 10.1126/science.1246775

[4] Shin, D. et al. Photon-efficient imaging with a single-photon camera. *Nat. Commun.* **7**, 12046 (2016). 27338821 10.1038/ncomms12046 PMC4931023

[5] McCarthy, A. et al. High-resolution long-distance depth imaging LiDAR with ultra-low timing jitter superconducting nanowire single-photon detectors. *Optica* **12**, 168–177 (2025).

[6] Li, Z. P. et al. Single-photon computational 3D imaging at 45 km. *Photonics Res.* **8**, 1532–1540 (2020).

[7] Li, Z. P. et al. Single-photon imaging over 200 km. *Optica* **8**, 344–349 (2021).

[8] O’Toole, M., Lindell, D. B. & Wetzstein, G. Confocal non-line-of-sight imaging based on the light-cone transform. *Nature* **555**, 338–341 (2018). 29513650 10.1038/nature25489

[9] Wu, C. et al. Non–line-of-sight imaging over 1.43 km. *Proc. Natl Acad. Sci. USA* **118**, e2024468118 (2021). 33658383 10.1073/pnas.2024468118 PMC7958383

[10] Giovannetti, V., Lloyd, S. & Maccone, L. Quantum-enhanced measurements: beating the standard quantum limit. *Science* **306**, 1330–1336 (2004). 15550661 10.1126/science.1104149

[11] Li, H. C. et al. Noise-tolerant LiDAR approaching the standard quantum-limited precision. *Light Sci. Appl.* **14**, 138 (2025). 40140381 10.1038/s41377-025-01790-5 PMC11947159

[12] Wang, F. F. et al. In vivo NIR-II fluorescence imaging for biology and medicine. *Nat. Photonics* **18**, 535–547 (2024).

[13] Rapp, J. et al. High-flux single-photon lidar. *Optica* **8**, 30–39 (2021).

[14] Peng, J. Y. et al. Boosting photon-efficient image reconstruction with a unified deep neural network. *IEEE Trans. Pattern Anal. Mach. Intell.* **45**, 4180–4197 (2023). 35994546 10.1109/TPAMI.2022.3200745

[15] Hong, Y. et al. Airborne single-photon LiDAR towards a small-sized and low-power payload. *Optica* **11**, 612–618 (2024).

[16] Fang, J. N. et al. Mid-infrared single-photon 3D imaging. *Light Sci. Appl.* **12**, 144 (2023). 37296123 10.1038/s41377-023-01179-2 PMC10256700

